# The prognostic role of nutrition risk score (NRS) in patients with metastatic or recurrent esophageal squamous cell carcinoma (ESCC)

**DOI:** 10.18632/oncotarget.20530

**Published:** 2017-08-24

**Authors:** Xia Zhou, Guo-qin Qiu, Wu-an Bao, Dan-Hong Zhang

**Affiliations:** ^1^ Department of Radiotherapy, Zhejiang Cancer Hospital, Hangzhou, Zhejiang, China; ^2^ Department of Radiation Therapy Zhejiang Cancer Hospital 38 Guangji Road, Hangzhou, Zhejiang, China

**Keywords:** esophageal squamous cell carcinoma, metastasis, nutrition, chemotherapy

## Abstract

The purpose of this study was to elucidate the prognostic value of nutritional risk score (NRS) in patients with metastatic or recurrent ESCC. A total of 187 patients who undergoing S1 based or paclitaxel based salvage chemotherapy were enrolled in this retrospective study. Nutritional status was evaluated by NRS. The relationship between NRS and clinicopathological variables and post-treatment outcomes were assessed by univariate and multivariate analysis. NRS was significantly associated with weight loss (P<0.001), BMI (P<0.001), chemotherapy regimens (P=0.038) and treatment response (P=0.013). The Kaplan-Meier survival curves indicated that patients with NRS ≥ 3 had worse overall survival (OS) compared to patients with NRS < 3 (P<0.001). Multivariable regression revealed that weight loss, NRS and treatment response were three prognostic factors (P<0.05). These results suggest that NRS is a promising indicator of poor prognosis in patients with metastatic or recurrent ESCC who received S1 based or paclitaxel based salvage chemotherapy.

## INTRODUCTION

Esophageal cancer remains the eighth most commonly diagnosed cancer worldwide. Although the five-year overall survival rate for patients with localized disease approaches 85% [1], the survival rate for metastatic or recurrent disease is only 5% [2]. Most patients are died of nutritional problem that leading to metabolic and physiological changes.

Esophageal squamous cell carcinoma (ESCC) is considered to be the predominant type in eastern Asia countries, with increased incidence recently [3]. Patients are often presented with obstructive symptoms, such as dysphagia and unintended weight loss. Furthermore, the psychological influence of cancer diagnosis and treatment can cause low mood or depression, which also reduce patients’ appetite [4]. Most patients could be affected by malnutrition during diagnosis, cancer treatment and follow-up. A consensus exists that weight loss is one of the most important criteria for malnutrition. Weight loss, poor life-style associated factor, is a common symptom in 60% of patients before diagnosis [5]. Significant weight loss resulted in systemic inflammation [6], higher rate of treatment complications and lower quality of life. Many studies reported that weight loss is associated with cancer recurrence in gastric cancer [7] and breast cancer [8]. However, weight loss alone does not identify the full effect of malnutrition on physical function [9] and is not a perfect prognostic factor [10]. Nutrition risk score (NRS) is a novel method for distinguishing high risk patients who will suffer from malnutrition and related with survival in gastric cancer [11]. Therefore, we were interested in that if this new method can be used in metastatic or recurrent ESCC patients who received palliative chemotherapy.

The aim of this study was investigate the association between NRS and other nutrition variables and the prognostic value of NRS in patients with metastatic or recurrent ESCC.

## RESULTS

### Patients’ characteristics

A total of 187 patients with metastatic or recurrent ESCC were analyzed. Baseline characteristics were presented in Table 1. Most patients were male (61.5%) with the median age of 61 years (range: 38-78 years). Most patients (66.8%, 125/187) had metastatic disease and 13 patients (7.0%) both had metastatic and recurrent disease. More than half of the patients (62.6%, 117/187) had experienced subtotal transthoracic esophagectomy and regional lymphadenectomy with curative intent, and other 70 patients (37.4%) had experienced radical radiotherapy. Most patients (57.8%) received S1 based chemotherapy and the remaining patients (41.2%) received paclitaxel based chemotherapy. All procedures performed in studies involving human participants were in accordance with the ethical standards of the institutional research committee. This study was approved by the institutional review board of the hospital. Informed consent was obtained from all patients included in the study.

**Table 1:** The nutrition risk score (NRS) and clinicopathological characteristics in 187 metastatic or recurrent ESCC

Variables	Patients (n)	NRS		P
		<3	≥3	
Gender				
Female	72	32 (44.4)	40 (55.6)	0.495
Male	115	57 (49.6)	58 (50.4)	
Age (Years)				
≤60	92	45 (48.9)	47 (51.1)	0.722
>60	95	44 (46.3)	51 (53.7)	
First line treatment				
Radiotherapy	70	33 (47.1)	37 (52.9)	0.924
Surgery	117	56 (47.9)	61 (52.1)	
Weight loss				
Yes	90	6 (6.7)	84 (93.3)	<0.001
No	97	83 (85.6)	14 (14.4)	
Tumor location				
Upper and middle	92	45 (48.9)	47 (51.1)	0.722
Lower	95	44 (46.3)	51 (53.7)	
Serum albumin level				
≤42.1	88	38 (43.2)	50 (56.8)	0.255
>42.1	99	51 (51.5)	58 (48.5)	
BMI				
≤20.4	95	30 (31.6)	65 (68.4)	<0.001
>20.4	92	59 (64.1)	33 (35.9)	
Failure type				
Metastasis	125	55 (44.0)	70 (56.0)	0.294
Recurrent	49	28 (57.1)	21 (42.9)	
Metastasis and recurrent	13	6 (46.2)	7 (53.8)	
Chemotherapy regimens				
S1	108	44 (40.7)	64 (59.3)	0.038
Paclitaxel	79	45 (57.0)	34 (43.0)	
Treatment response				
CR+PR	67	40 (59.7)	27 (40.3)	0.013
SD+PD	120	49 (40.8)	71 (59.2)	
Second line treatment				
Yes	62	30 (48.4)	32 (51.6)	0.835
No	124	58 (46.8)	66 (53.2)	

### Relationship between NRS and clinical features

According to NRS, 52.4% (n=98) had a score ≥3. The median NRS for all patients was 3. NRS was significantly associated with weight loss (P<0.001), BMI (P<0.001), chemotherapy regimens (P=0.038) and treatment response (P=0.013), whereas there was no significant association between gender (P=0.495), age (P=0.722), first line treatment (P=0.924) and tumor location (P=0.722). Correlation of NRS with BMI, weight loss, serum albumin level, hemoglobin and tumor markers are presented in Table 2. Spearman's correlation revealed that NRS had a positive correlation with weight loss (P<0.001) and serum albumin level (P<0.001).

**Table 2:** Correlation of NRS with nutritional variables and tumor markers

Variables	NRS	
	Correlation	P
BMI	-0.085	0.247
Weight loss	0.743	<0.001
Serum albumin level	-0.396	<0.001
Hemoglobin	-0.095	0.196
Red blood cell count	-0.011	0.884
CEA	0.112	0.140
CA125	-0.057	0.475
CA199	-0.039	0.602
CA724	-0.084	0.368

### Association of NRS with survival

**Figure 1:**
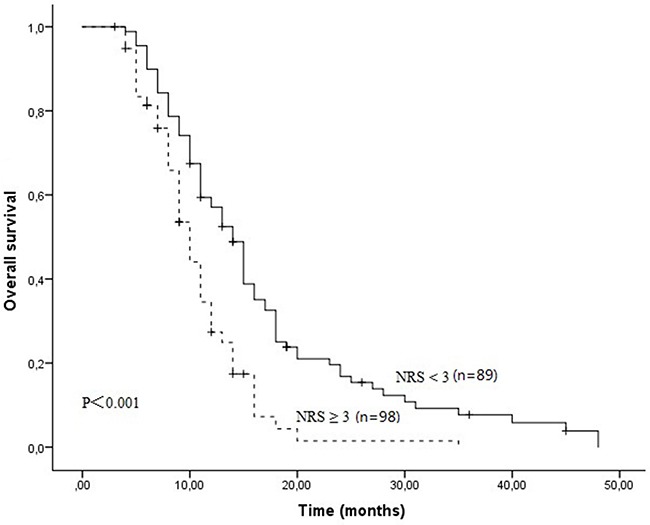
The Kaplan-Meier survival curves indicated that patients with NRS ≥ 3 had worse OS compared to patients with NRS < 3 (11.0 months Vs 14.0 months, P<0.001)

**Figure 2:**
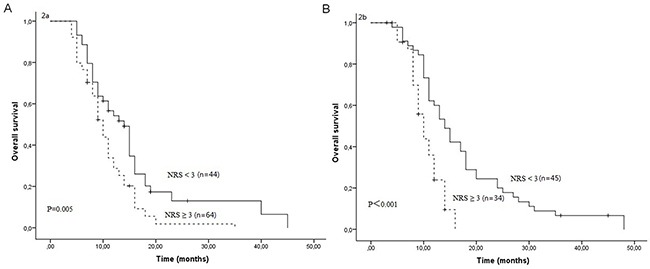
Overall survival curves between NRS ≥ 3 and NRS < 3 in patients who received S1 based chemotherapy **(A)** and in patients who received paclitaxel based chemotherapy **(B)**.

**Figure 3:**
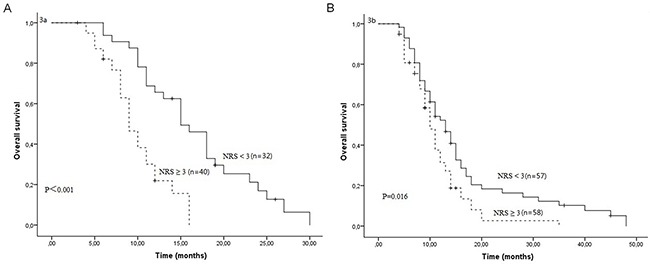
Overall survival curves between NRS ≥ 3 and NRS < 3 in female patients **(A)** and in male patients **(B)**.

After a median follow-up duration of 23 months, the estimate 1-year and 2-year overall survival rates in all patients were 42.3% and 9.4%, respectively. One hundred and sixty-four patients died due to tumor progression or malnutrition. Weight loss, treatment response and NRS were significantly related with OS (Table 3). The Kaplan-Meier survival curves indicated that patients with NRS ≥ 3 had worse OS compared to patients with NRS < 3 (P<0.001, Figure 1). In further analyses, NRS ≥ 3 was significantly associated with shorter OS for patients who received S1 based chemotherapy (P=0.005, Figure 2A) and patients who received paclitaxel based chemotherapy (P<0.001, Figure 2B). One-year survival probability was 28.7% for NRS ≥ 3 patients versus 54.2% for NRS < 3 patients with S1 based chemotherapy, and 23.9% for NRS ≥ 3 patients versus 60.0% for NRS < 3 patients with paclitaxel based chemotherapy. Similarly, NRS ≥ 3 was significantly associated with shorter OS for female patients (P<0.001, Figure 3A) and male patients (P=0.016, Figure 3B).

**Table 3:** Prognostic factors for OS by univariate analysis for patients with metastatic or recurrent ESCC (n=187)

Variables	Patients (n)	Median OS (months)	1-year OS rate (%)	2-year OS rate (%)	P
Gender					
Female	72	12.0	48.0	9.2	0.956
Male	115	12.0	46.1	12.2	
Age (Years)					
≤60	92	14.0	53.9	13.0	0.314
>60	95	11.0	40.3	8.8	
First line treatment					
Radiotherapy	70	11.0	46.1	10.3	0.711
Surgery	117	11.0	41.7	8.8	
Weight loss					
Yes	90	13.0	38.9	4.2	0.012
No	97	11.0	53.4	17.0	
Tumor location					
Upper and middle	92	11.0	42.1	7.5	0.203
Lower	95	13.0	51.3	14.1	
Serum albumin level					
≤42.1	88	11.0	43.2	11.6	0.642
>42.1	99	14.0	50.2	10.5	
BMI					
≤20.4	95	11.0	42.2	9.4	0.789
>20.4	92	13.0	51.6	13.2	
Tumor length					
≤5 cm	46	13.0	50.8	11.5	0.845
>5 cm	141	11.0	45.6	10.0	
Tumor					
Metastasis	125	11.0	42.9	9.7	0.234
Recurrent	49	12.0	48.1	12.2	
Metastasis and recurrent	13	10.0	15.4	0	
Chemotherapy regimens					
S1	108	11.0	43.3	5.9	0.071
Paclitaxel	79	12.0	46.5	16.7	
Treatment response					
CR+PR	67	16.0	75.5	26.1	<0.001
SD+PD	120	10.0	29.9	2.1	
NRS					
< 3	89	14.0	57.1	16.8	0.002
≥3	98	11.0	36.7	5.4	

Then we performed multivariable regression using a Cox proportional hazards models revealed that weight loss, NRS and treatment response were three prognostic factors in patients with metastatic or recurrent ESCC (P<0.05). Patients with NRS ≥ 3 had an elevated risk of death compared to those with NRS < 3. The hazard ratio was 2.14 (95% confidence interval [CI] 1.25–3.68) for death (Table 4).

**Table 4:** Prognostic factors for OS by multivariate Cox regression analysis for patients with metastatic or recurrent ESCC

Variables	Retrospective cohort			Validation cohort		
	HR	95% CI	P	HR	95% CI	P
Weight loss (Yes Vs No)	0.54	0.31-0.94	0.030	0.96	.65-1.42	0.837
N stage (N0 Vs N1)	0.79	0.58-1.08	0.144	0.74	0.51-1.07	0.104
Treatment response (SD+PD Vs CR+PR)	3.29	2.25-4.81	<0.001	1.51	1.02-2.26	0.044
NRS (≥3 Vs < 3)	2.14	1.25-3.68	0.006	1.58	1.07-2.34	0.024

### NRS in the validation cohort

**Figure 4:**
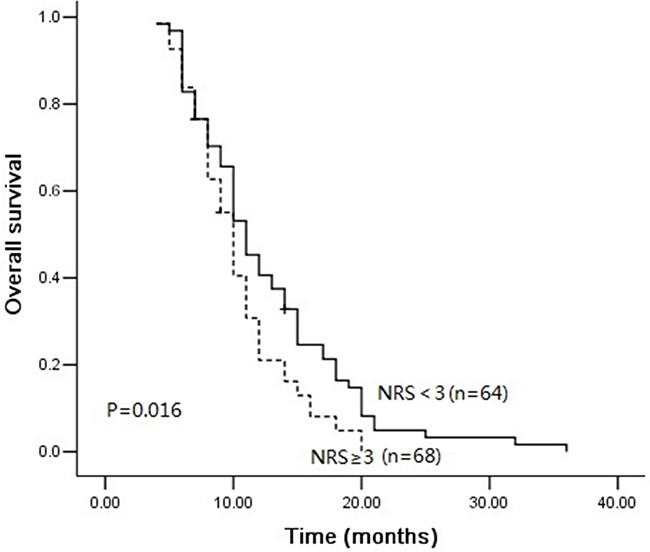
Overall survival curves between NRS ≥ 3 and NRS < 3 in validation group

When the cutoff value of 3 was used in the validation cohort, 68 (51.5%) of the ESCC patients were observed to have NRS ≥ 3. The age distribution, tumor location, treatment response, and NRS were well balanced between the two patients’ cohorts (P>0.05). The patients in the validation cohort with NRS ≥ 3 exhibited decreased OS (P=0.016) compared with the patients who had NRS < 3 (Figure 4). The multivariate COX regression analysis showed that NRS ≥ 3 and treatment response were independent predictors (Table 4).

## DISCUSSION

In this present study, we investigated NRS in metastatic or recurrent ESCC and its correlation with prognosis. Our data demonstrated that NRS was significantly associated with weight loss and serum albumin level. NRS was an independent prognostic factor related with overall survival in metastatic or recurrent ESCC. As predicted, the OS was better for patients with NRS < 3 than for patients with NRS ≥ 3.

Currently, the TNM staging system is a standard method to predict patients’ prognosis. However, clinicians need an accurate tool to manage the treatment and predict survival when patients developed distant metastasis or local-regional recurrence. Tumor markers, such as CEA, SCC and CYFRA2-11, are not precisely associated with outcome in metastatic or recurrent ESCC patients [12]. In our study, tumor markers (CEA, CA125, CA199 and CA724) were not associated with overall survival according to univariate analysis (P>0.05). Several studies reported that the nutritional and immunologic conditions of patients could influence the post-operative complications and outcome with cancer [13–15]. NRS, calculated based on patients’ weight loss and BMI, is a significant predictor for malnutrition according to Cox et al’ report [16]. The nutritional status was also shown recently to be an indicator for complications in patients with esophageal cancer [17, 18]. Early nutritional support could significantly reduce severe complications which related with high morbidity, such as pulmonary complications and anastomotic leakage [19]. Although past studies have shown important findings for NRS, its clinical significance has not yet been clarified in patients with metastatic or recurrent ESCC. In the present study, NRS was significantly related with treatment response and multivariate analysis revealed that NRS, not serum tumor markers and BMI, was an independent indicator for prognosis. The combination of TNM stage and NRS may be a more effective tool for predicting treatment response and outcome in patients with metastatic or recurrent ESCC. Physicians should investigate patients’ nutritional status, not only based on serum albumin level or BMI, but also on NRS. Patients with higher NRS (NRS≥3) should receive close post-treatment follow-up.

Another important result of this study was that in patients with metastatic or recurrent ESCC, salvage chemotherapy with paclitaxel-based regimens could slightly improve overall survival (P=0.071). In Yang et al’ report, chemotherapy with paclitaxel-based regimens demonstrated higher efficacy with less toxicity in patients with ESCC compared with the fluorouracil based regimens [20]. The median PFS in patients received paclitaxel-based regimens were significantly longer than in patients received S1-based regimens (13.0 m Vs 6.5 m, P=0.034). In our study, the estimate 2-year OS rates were 16.7% and 5.9% in patients received paclitaxel-based regimens and S1-based regimens. It seems in salvage chemotherapy using paclitaxel-based regimens was a promising treatment in patients with metastatic or recurrent ESCC. However, further large scale randomized clinical trials are needed to confirm this result.

There are several limitations of this study. The major limitation is that the information of post-treatment local recurrence or metastasis was insufficient. One of the least convincing things in this study are lack the data of disease free survival, although overall survival is the standard indicator in the cancer prognosis study. Another limitation is the number of study samples was relatively small. Also, some nutritional parameters, such as TSF (triceps skin fold), MAC (mid-arm muscle circumference), HGS (handgrip strength) and MAMA (mid-arm muscle area), were insufficient. Therefore, we cannot compare NRS with other nutritional scores, like sPG-SGA [13].

The results of this study suggest that NRS are strongly related with BMI, weight loss and treatment response in patients with metastatic or recurrent ESCC. Additionally, NRS can be used as a possible marker to predict overall survival. Patients with NRS ≥ 3 had an elevated risk of death compared to those with NRS < 3. The significance of these results merit further validation in a larger cohort of patients.

## MATERIALS AND METHODS

### Patients

This study in patients with metastatic or recurrent ESCC was conducted at Zhejiang cancer hospital, Hangzhou, China. A total of 187 patients were enrolled in this study. Only patients with histologically confirmed diagnosis of ESCC were included. Patients with the following characteristics were excluded from our study: patients showed a severe functional impairment of vital organs who cannot tolerate chemotherapy; those whose expectancy life less than 3 months. The nutritional status evaluation was performed by two independent investigator at the first outpatient visit after verified metastatic or recurrent ESCC diagnosis. Clinical details such as gender, age, tumor histopathology, tumor site and TNM stage were collected from hospital records. This study was approved by the institutional review board of the hospital. All patients provided informed consent before treatment.

We then used the validation cohort to test the result in predicting prognosis in patients with metastatic or recurrent ESCC. The validation cohort data were collected in the same hospital of patients who received salvage chemotherapy between October 2016 and March 2017. Finally, we identified 132 cases in the validation cohort that fit the inclusion criteria.

### Nutritional assessment

Nutritional assessment was evaluated by nutrition risk score (NRS) [11, 13, 21]. NRS consisted of the combination of weight loss, body mass index (BMI), age and severity of disease. The final score ranges from 0-7. According to the previous report, NRS ≥3 was considered high nutritional risk [13]. On admission to our department, the patients’ height and weight were documented and detail information about weight loss was obtained using a structured questionnaire. BMI was calculated using the well-known formula. Weight loss was defined as exceeding five percent of habitual body weight in the preceding three months or ten percent in the preceding six months. Blood samples were taken from each patient routinely before treatment to measure serum albumin, hemoglobin and tumor markers (CEA, CA125 and CA199).

### Treatment

Chemotherapy consisted of four to six cycles of S1 based chemotherapy or paclitaxel based chemotherapy according to patients’ performance status score and their preference. If patient's PS score=1 or 2, S1 based chemotherapy was recommended, otherwise paclitaxel based chemotherapy was recommended. Chemotherapy was stopped when there was unacceptable toxicities and disease progression. Best support care (BSC) was given to all patients, including pain management, nutritional treatment, esophageal dilation or stent placement and blood product transfusions.

### Assessment and follow-up

Tumor assessment was performed 6 weeks after treatment or earlier in cases of clinical suspicion of progression. The objective response to treatment was defined using the Response Evaluation Criteria in Solid Tumors (RECIST 1.1) [22]. For this analysis, patients with complete response (CR) or partial response (PR) were classified as responders, and those with stable disease (SD) or progressive disease (PD) were defined as non-responders.

All patients received standardized follow-up at a 2-month interval for the first 2 years after operation, a 6-month interval in the third year and yearly thereafter. Evaluation comprised physical examination, complete blood count, chest computed tomography, esophagogram and abdominal ultrasound.

### Statistical analysis

Overall survival (OS) was defined as the time interval from the initial event (diagnosis) to the death or censoring. The chi-square test was performed to evaluate the association between the clinicopathological variables and NRS. The correlations between NRS with BMI, weight loss, pretreatment albumin, pretreatment hemoglobin, pretreatment red blood cell count and tumor markers (CEA, CA125, CA199 and CA724) were estimated by linear correlation analysis. Survival curves were estimated by the univariate Kaplan-Meier method. The log-rank test was applied to check the significant differences in the curves among groups. Furthermore, we used the Cox proportional hazards model for multivariate analysis. All statistical calculations were performed with SPSS 21.0 for Windows (Chicago, IL). Two-sides P values of < 0.05 were considered statistical significance.
